# Menstrual Cycle Management and Period Tracker App Use in Millennial and Generation Z Individuals: Mixed Methods Study

**DOI:** 10.2196/53146

**Published:** 2024-10-10

**Authors:** Minji Hong, Vasuki Rajaguru, KyungYi Kim, Suk-Yong Jang, Sang Gyu Lee

**Affiliations:** 1 Department of Medical Device Engineering and Management College of Medicine Yonsei University Seoul Republic of Korea; 2 Department of Healthcare Management Graduate School of Public Health Yonsei University Seoul Republic of Korea; 3 Department of Preventive Medicine College of Medicine Yonsei University Seoul Republic of Korea

**Keywords:** menstruation, dysmenorrhea, period tracker app, menstrual cycle management, health care application, millennial, Gen Z, mobile phone

## Abstract

**Background:**

Menstruation is a physical symptom that occurs in women of reproductive age. It has a significant impact on the daily life and health of women when their academic and social activities are most active. Since many women experience difficulties in daily life because of premenstrual syndrome and dysmenorrhea, it is important to identify, prepare for, and manage the menstrual cycle in advance.

**Objective:**

This study aimed to investigate the relationship between menstruation-related experiences and the use of mobile-based period tracker apps by millennial and generation Z (gen Z) individuals. The objectives of this study are to investigate (1) menstrual cycle management, (2) factors affecting app usage (3) factors affecting cycle management, and (4) motivators and barriers to using period tracker apps, in millennial and gen Z women.

**Methods:**

A mixed methods design was used for this study. The participants were young women aged 20-39 years and recruited via the Ovey application. Data were collected through surveys and focus group interviews. The survey was conducted among 700 women, and 8 of them participated in the focus group interviews.

**Results:**

In total, 431 (62.3%) participants used period tracker apps primarily to predict their next menstrual cycle. Factors affecting app usage included childbirth experience (odds ratio [OR] 0.475, *P*<.05), number of dysmenorrhea symptoms (OR 1.136, *P*<.05), and cycle management level (OR 2.279, *P*<.001). Additionally, education level (OR 1.122, *P*<.05 [university level compared high school level]) and the number of dysmenorrhea symptoms (OR 1.024, *P*<.05) showed a positive association with the cycle management level. However, childbirth experience (OR 0.902, *P*<.05) and period irregularity (OR 0.929, *P*<.001) were negatively associated with the cycle management level.

**Conclusions:**

Period tracker apps are becoming the new normal among millennials and gen Z individuals for managing their menstrual cycles. The use of a period tracker app empowers women by helping them gain a better understanding of their bodies, ultimately enhancing their social, academic, and health-related lives. Improving the accuracy and literacy of the app is an ongoing task for period-tracking apps, and it is important to provide added value tailored to users’ needs. Therefore, the findings of this study should be considered when designing or upgrading period tracker apps to facilitate the adoption of digital technology among young women, thereby promoting their overall well-being and reproductive health.

## Introduction

Menstruation is a physical symptom that occurs in women of reproductive age and can bring pain or cramps during the menstrual period (hereafter dysmenorrhea) and premenstrual syndrome (PMS), which includes symptoms experienced before the menstrual period. Menstruation significantly impacts women's daily lives when their academic and social activities are most active [1-5], with 85.4% experiencing dysmenorrhea, and 38.4% reporting reduced activity due to menstrual pain [6].

Period tracker apps (PTA), introduced in 2013, offer a new way to manage menstrual cycles, helping over 50 million women globally to track their menstrual cycles, mood changes, and symptoms [7]. PTA users may better understand these patterns, control their moods, and improve their general well-being with the help of PTA.

The main purpose of PTA is to provide information on the number of days left until the next cycle and ovulation. Most apps provide additional features such as cycle-related mood changes, pain and sleep pattern records, and statistical information about average cycles [8]. These apps provide valuable data on cycle patterns, which can help women manage their menstruation more effectively. In the United Kingdom, 38% of women use PTAs mainly for cycle monitoring (72%), pregnancy planning (34%), and obtaining information on infertility treatment (12%) [9].

While PTAs are not specifically designed to treat PMS or dysmenorrhea, studies suggest that they may help alleviate these symptoms [10]. Studies have been focused on the experience of using PTAs or PTA data to understand the experience and app use [7,8,11], feasibility of apps [10-13], effectiveness of period prediction [9], and evaluate the apps from various aspects, such as the information and functions they provide [14-23]. In addition, the association between dysmenorrhea and PMS compared the characteristics of PTA users and nonusers was conducted [9,24].

While numerous studies have focused on PTAs, there remains a significant gap in understanding how these apps are integrated into women's daily lives and how user characteristics influence app usage.

This study aims to explore the relationship between menstruation-related experiences and PTA use among millennials and generation Z (gen Z), who are adept with smartphones and digital devices. This study proposes future implications for the development of women's health mobile apps by providing basic research data on menstrual experiences and management in millennial and gen Z women. The research addresses the following research questions: (1) What is the menstrual cycle management and app usage status of millennials and gen Z? (2) Which social factors and menstrual-related experiences affect PTA use in millennials and gen Z? (3) Which social factors and menstrual-related experiences influence their cycle management level in millennials and gen Z? (4) What are the motivators and barriers to using PTAs in millennials and gen Z?

## Methods

### Study Design

**Figure 1:**
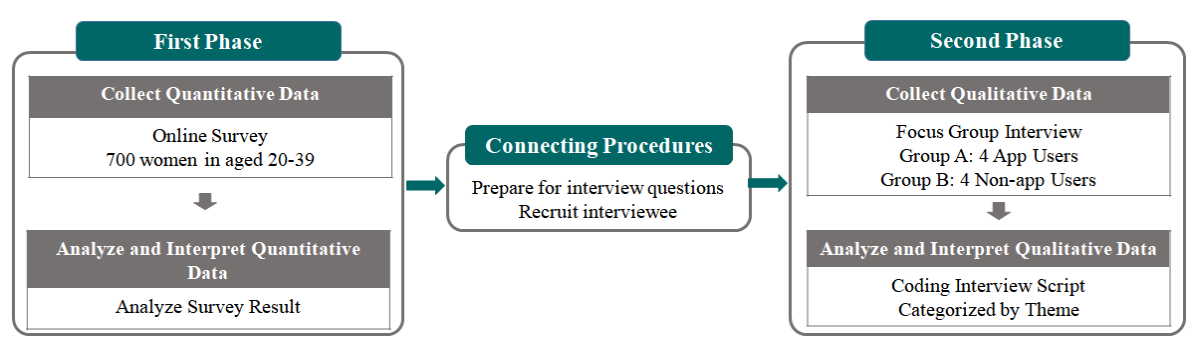
Study design.

A mixed methods approach was used in this study. A quantitative survey was first conducted, and qualitative research data were collected through interviews with some of the survey respondents. This explanatory sequential design has the advantage of detailing the quantitative results using a qualitative method [25,26]. The detailed research design is shown in Figure 1. It includes a first phase to collect and analyze quantitative data, a connecting procedure between the first and second phases, and a second phase to collect and analyze qualitative data. Quantitative and qualitative data were linked using panel codes. The study design was planned by referring to the mixed methods research guideline published by the National Institutes of Health [27].

### Ethical Considerations

The study protocol was reviewed and approved by the institutional review board of Severance Hospital (4-2023-0097). This approval ensures that the study complies with ethical standards and regulations for research involving human subjects.

Participants responded to the survey only after reading the study's purpose and agreement details provided through the mobile app. Given the nature of the survey, which involved minimal risk and was conducted via a mobile application, the requirement for written consent was waived by the institutional review board. The comprehensive survey information sheet for participants is presented in Table S1 in Multimedia Appendix 1**.**

To protect the privacy and confidentiality of participants, all collected data were anonymized. Identifiable information was not collected, ensuring that individual responses could not be traced back to any participant. Data were securely stored and only accessible to the research team. Additionally, any published results do not include information that could potentially identify participants.

Survey participants received 500 credits, equivalent to 500 Korean won, as compensation for their participation. Additionally, participants in the focus group interviews (FGIs) received 60,000 credits, equivalent to 60,000 Korean won. These incentives were provided to recognize the time and effort contributed by the participants while ensuring transparency and fairness in the compensation process.

### Participants

The target population of this study was women aged 20-39 years. Participants were recruited from “Open survey” (Ovey), which is a platform that can provide surveys and pay compensation and has 830,000 voluntarily registered panels. Among those who met the criteria, participants were randomly recruited through an app push, and only those who read and agreed with the explanation participated in the survey. The target number of participants was 700, including 350 females aged 20-29 years and 350 females aged 30-39 years, respectively. We defined gen Z as those aged ≤27 years and millennials as those aged 28-39 years at the time of response.

### Survey

The survey was conducted on the web in March 2023, using the Ovey application. The web-based survey comprised 29 multiple choices, Likert, open-ended questions. Questions were related to respondents’ menstrual experience, menstrual cycle management behavior, usage of PTAs, and thoughts about PTAs. Depending on the preceding response, the number of questions to be answered could change; the participants responded to 15-26 questions.

The questions in the survey were developed based on previous research and the specific objectives of this study. We conducted pilot testing with a sample of 20 women. Based on the feedback and comments received during the pilot test, we revised the questionnaire to improve clarity and construction. On average, it took participants approximately 5 minutes to complete the questionnaire. We designed the survey to be concise to minimize the burden on respondents while still collecting essential information. The reporting of the survey was guided by the CHERRIES (Checklist for Reporting Results of Internet E-Surveys; Table S2 in Multimedia Appendix 2).

### Interviews

Among the participants who responded to the survey, those who were available for FGIs were recruited. A total of 8 people were interviewed by selecting 4 from PTA users and 4 from nonusers. In May 2023, FGIs were conducted twice on the web, and each interview lasted approximately 60 minutes. The first and second interviews were conducted among 4 app users and 4 nonusers, respectively. FGIs were recorded on Zoom and transcribed automatically using Clova Note from Naver. The questionnaire for the FGI can be found in Table S3 in Multimedia Appendix 3 of the FGI guide. The reporting of the focus groups was guided by the COREQ (consolidated criteria for reporting qualitative research; Table S4 in Multimedia Appendix 4)

### Data Analyses

Data analysis was performed in 3 parts. First, we gathered data from app users’ information. Second, a survey was conducted to determine women’s menstrual experiences, app use, and tracking practices. Cycle management level is an indicator used to represent the degree to which women manage their menstrual cycles, and it is divided into 6 levels. Level 0=No record; 1=Record sometimes; 2=Record regularly; 3=Record and predict next cycle; 4=Record, predict, and reference plans; and 5=Record, predict, and change plans. Logistic regression and a generalized linear model (with log link and gamma distribution) were used to analyze the survey data. Logistic regression was used to determine the association between user characteristics and app usage, and gamma regression was used to identify factors influencing cycle management level. We examined collinearity of variables and the results of the collinearity test indicated that the VIF values for all variables were confirmed to be 2.7 or below. Third, the FGI results of the verbalized transcripts were coded, and the transcriptions were analyzed using inductive thematic analysis. Two authors independently coded each record after reviewing the encoded transcripts, implying a significant user perspective. The team then repeatedly incorporated and altered the codes to create a set of themes. Finally, the themes were identified and described using illustrative participant comments. The collected data were quantitatively analyzed using STATA (version 17.0; StataCorp), and qualitative data were analyzed using NVivo software (version 14; QSR International).

## Results

### General Characteristics

A total of 700 responses were collected, and 8 responses which gave inconsistent answers were excluded from the screening process. Table 1 presents the demographics of the survey respondents. Most of these individuals were at the university level (n=450, 73.9%) in education, were employed (n=403, 58.2%), and were unmarried (n=475, 68.6%). Millennials made up 64% (n=443) of the total respondents, with the majority aged 30-34 years (n=190, 42.9%).

**Table 1:** Demographics of the total 692 survey respondents.

Characteristics			Overall		Generation Z		Millennials
Total, n (%)			692 (100.0)		249 (36.0)		443 (64.0)
Age in years, mean (95% CI)			29.9 (29.6-30.3)		24.3 (24.1-24.6)		33.1 (32.8-33.4)
**Age groups (years), n (%)**							
	20-24	119 (17.2)		119 (47.8)		—^a^	
	25-29	226 (32.7)		130 (52.2)		96 (21.6)	
	30-34	190 (27.5)		—		190 (42.9)	
	35-39	157 (22.7)		—		157 (35.5)	
**Education^b^, n (%)**							
	High school	56 (8.1)		18 (7.2)		38 (8.5)	
	College level	138 (19.9)		40 (16.1)		98 (22.2)	
	University level	450 (65)		184 (73.9)		266 (60)	
	Master/doctoral	48 (6.9)		7 (2.8)		41 (9.2)	
**Occupation, n (%)**							
	Student	107 (15.5)		100 (40.2)		7 (1.6)	
	Employee	403 (58.2)		101 (40.6)		302 (68.2)	
	Homemaker	69 (10)		2 (0.8)		67 (15.1)	
	Self-employee	19 (2.7)		2 (0.8)		17 (3.8)	
	Others	38 (5.5)		14 (5.6)		24 (5.4)	
	No occupation	56 (3.8)		30 (12)		26 (5.9)	
**Marriage, n (%)**							
	Unmarried	475 (68.6)		237 (95.2)		238 (53.7)	
	Married	217 (31.4)		12 (4.8)		205 (46.3)	

^a^Not applicable.

^b^Education level includes both students and graduates.

### Menstruation Experience and Cycle Management

The results of the survey on menstrual experiences and behaviors are presented in Table 2. Approximately half of the millennial and gen Z individuals reported a menarche age between 12 and 13 years. The most frequently reported average period length was 27-29 days (n=257, 36.9%), followed by 30-32 days (n=160, 23%), and 505 out of 692 (75.3%) had regular or relatively regular menstrual cycles. The proportion of those who reported irregular cycles (including relatively irregular cycles) was higher among gen Z than individuals than among millennials. A total of 68 respondents reported having severe PMS, and half of the respondents indicated that they experience strong or severe PMS. Regarding dysmenorrhea, more than half of both millennial and gen Z individuals experienced severe or strong period cramps. Regarding gynecologist visits over the past 3 years, excluding visits during pregnancy or childbirth, 445 (63.8%) respondents had never visited the clinic. Participants’ regularity in recording their period duration and date was evaluated by the period cycle management level. In total, 614 (88.7%) respondents routinely recorded their menstrual cycles. A total of 431 (62.3%) respondents reported using PTAs to record and track their menstrual cycles, with no significant difference between millennials and gen Z in PTA use.

**Table 2:** Survey participants menstrual experience and behavior.

Category		Overall	Generation Z	Millennials
Total, n (%)		692 (100)	249 (100)	443 (100)
**Menarche, n (%)**				
	Underage 12	114 (16.4)	48 (19.3)	65 (14.7)
	12-13	343 (49.2)	112 (45)	231 (52.1)
	14-16	185 (26.5)	68 (27.3)	117 (26.4)
	Over 16	50 (7.2)	21 (8.4)	29 (6.5)
**Period cycle, n (%)**				
	Do not know	19 (2.7)	8 (3.2)	11 (2.5)
	Less than 20 days	9 (1.3)	4 (1.6)	5 (1.1)
	21-23 days	42 (6)	19 (7.6)	23 (5.2)
	24-26 days	77 (11)	24 (9.6)	53 (12)
	27-29 days	257 (36.9)	79 (31.7)	178 (40.2)
	30-32 days	160 (23)	59 (23.7)	101 (22.8)
	33-40 days	93 (13.3)	43 (17.3)	50 (11.3)
	More than 41 days	35 (5)	13 (5.2)	22 (5)
**Cycle Regularity, n (%)**				
	Regular	202 (29)	66 (26.5)	135 (30.5)
	Relatively regular	303 (43.5)	90 (36.1)	211 (47.6)
	Relatively irregular	119 (17.1)	61 (24.5)	58 (13.1)
	Irregular/amenorrhea	73 (10.5)	32 (12.9)	39 (8.8)
**PMS^a^ severity, n (%)**				
	None	10 (1.4)	4 (1.6)	6 (1.4)
	Mild	63 (9)	28 (11.2)	35 (7.9)
	Moderate	266 (38.2)	101 (40.6)	165 (37.2)
	Strong	285 (40.9)	96 (38.6)	189 (42.7)
	Severe	68 (9.8)	20 (8)	48 (10.8)
**Dysmenorrhea severity, n (%)**				
	None	9 (1.3)	4 (1.6)	5 (1.1)
	Mild	82 (11.8)	28 (11.2)	54 (12.2)
	Moderate	236 (33.9)	84 (33.7)	152 (34.3)
	Strong	289 (41.5)	111 (44.6)	178 (40.2)
	Severe	76 (10.9)	22 (8.8)	54 (12.2)
**Visit OBGY^b^ in 3 years^c^, n (%)**				
	None	445 (63.8)	162 (65.1)	283 (63.9)
	1-2 times	171 (24.5)	62 (24.9)	109 (24.6)
	3-5 times	51 (7.3)	16 (6.4)	35 (7.9)
	6-9 times	14 (2)	4 (1.6)	10 (2.3)
	More than 10 times	11 (1.6)	5 (2.0)	6 (1.4)
**Cycle management level, n (%)**				
	No record	25 (3.6)	12 (4.8)	13 (2.9)
	Record sometimes	53 (7.6)	24 (9.6)	29 (6.5)
	Record regularly	235 (33.7)	86 (34.5)	149 (33.6)
	Record and predict next cycle	151 (21.7)	44 (17.7)	107 (24.2)
	Record, predict cycle, and reference plans	150 (21.5)	47 (18.9)	103 (23.3)
	Record, predict cycle, and change plans	78 (11.2)	36 (14.5)	42 (9.5)
**Cycle management method, n (%)**				
	No record	25 (3.6)	12 (4.8)	13 (2.9)
	Calendar/diary (Paper)	75 (10.8)	23 (9.2)	52 (11.7)
	Calendar/diary (Digital)	161 (23.3)	56 (22.5)	105 (23.7)
	PTA^d^	431 (62.3)	158 (63.5)	273 (61.6)

^a^PMS: premenstrual syndrome.

^b^OBGY: obstetrics and gynecology.

^c^Number of visits to obstetricians and gynecologists related to menstruation, such as menstrual irregularities. Visits owing to pregnancy or childbirth were excluded.

^d^PTA: Period tracker apps.

### Factors Affecting PTA Use

PTA user and nonuser characteristics are shown in Table 3. Chi-square analysis was used to analyze the differences between the 2 groups according to several predictors. App users and nonusers showed statistically significant differences in marital status (*P*=.006), with unmarried individuals being more likely to use PTA. Childbirth experience significantly impacts PTA app usage (*P*<.001), with those who have not experienced childbirth being more likely to use the app. Logistic regression was used to determine factors that affect PTA use; marriage, childbirth experience, number of PMS symptoms, and number of dysmenorrhea symptoms were found to be statistically significant (Table 4). As an important social factor, childbirth experience was found to affect PTA use. In women with childbirth experience, the likelihood of using a PTA decreased by 52.5% compared to women without childbirth experience. To estimate the levels of PMS and dysmenorrhea, we used 3 indicators: perceived severity, effect on daily life, and number of symptoms. Among these indicators, only the number of dysmenorrhea symptoms showed statistical significance. A higher number of dysmenorrhea symptoms is significantly associated with increased PTA usage (*P*=.04). Each additional symptom increases the likelihood of using PTA by approximately 13.6%. The number of dysmenorrhea symptoms and cycle management level were positively associated with app use. Individuals with higher cycle management levels are significantly more likely to use PTA (*P*<.001). They are more than twice as likely to use PTA compared to those with lower cycle management levels.

**Table 3:** PTA^a^ users and nonuser’s characteristics.

Predictor				Nonuser		App user		*P* value^b^	
Total, n (%)				261 (37.7)		431 (62.3)		—^c^	
**Social factor**									
	**Generation, n (%)**								.63
		Millennial	170 (38.4)		273 (61.6)				
		Generation Z	91 (37)		158 (63.5)				
	**Marriage, n (%)**								.006
		Married	98 (45.2)		119 (54.8)				
		Unmarried	163 (34.3)		312 (65.7)				
	**Childbirth experience, n (%)**								<.001
		Yes	80 (52.6)		72 (47.0)				
		No	181 (33.5)		359 (66.5)				
	**Education level^d^, n (%)**								.54
		High school graduate	24 (42.9)		32 (57.1)				
		College level	53 (38.4)		85 (61.6)				
		University level	170 (37.8)		280 (62.2)				
		Graduate school level	14 (29.2)		34 (70.8)				
**Menstrual experience**									
	**Period regularity, n (%)**								.64
		Regular	68 (33.8)		133 (66.2)				
		Relatively regular	119 (39.5)		182 (60.5)				
		Relatively irregular	44 (37.0)		75 (63.0)				
		Irregular	19 (44.2)		24 (55.8)				
		Amenorrhea	11 (39.3)		17 (60.7)				
	**PMS severity, n (%)**								.92
		None	4 (40.0)		6 (60.0)				
		Mild	22 (34.9)		41 (65.1)				
		Moderate	99 (37.2)		167 (62.8)				
		Strong	107 (37.5)		178 (62.5)				
		Severe	29 (42.6)		39 (57.4)				
	**Dysmenorrhea severity, n (%)**								.67
		None	4 (44.4)		5 (55.6)				
		Mild	25 (30.5)		57 (69.5)				
		Moderate	93 (39.4)		143 (60.6)				
		Strong	111 (38.4)		178 (61.6)				
		Severe	28 (36.8)		48 (63.2)				

^a^PTA: Period tracking app.

^b^Derived from Chi-square test.

^c^Not applicable.

^d^Education level includes both students and graduates.

**Table 4:** Results of statistical analyses using logistic regression to determine likelihood of period tracking app (PTA) usage.

Variables				Crude OR^a^		*P* value^a^		95% CI^a^		Adjusted OR^b^		*P* value^b^		95% CI^b^	
**Social Factor**															
	Childbirth Experience			0.454		<.001		0.315-0.654		0.475		.04		0.237-0.950	
	**Education level**														
		Baseline (High school graduate)	—^c^		—		—		—		—		—		
		College level	1.203		.57		0.640-2.260		1.021		.96		0.479-2.178		
		University level	1.235		.46		0.704-2.168		0.964		.92		0.492-1.891		
		Graduate school level	1.821		.15		0.805-4.123		2.177		.12		0.825-5.742		
**Menstrual experience**															
	Period irregularity			0.923		.07		0.796-1.071		1.096		.35		0.906-1.325	
	Effect of PMS^d^ on daily life			1.040		.11		0.853-1.268		0.839		.36		0.577-1.219	
	Effect of dysmenorrhea on daily life			1.110		.10		0.921-1.338		0.935		.71		0.653-1.337	
	Number of PMS symptoms			1.225		.06		1.110-1.351		1.100		.18		0.957-1.264	
	Number of dysmenorrhea symptoms			1.209		.05		1.117-1.308		1.136		.04		1.008-1.280	
	Cycle management level			2.209		.18		1.889-2.582		2.279		<.001		1.919-2.707	

^a^Crude odds ratio value from univariate model.

^b^Multivariate model. Adjusted odds ratio using the following predictors: age, marriage, menarche age, area group, visit obstetrics and gynecology in 3 years and occupation.

^c^Not applicable.

^d^PMS: premenstrual syndrome.

### Factors Affecting Cycle Management Level

Two models were used to analyze the factors affecting cycle management level (Table 5). As shown in Table 2, cycle management level was categorized into 6 levels, from “no record” (level 0) to “record, predict cycle, and change plan” (level 5). A larger value indicates a more active attitude in cycle management level. Model 1 was established to investigate social factors such as childbirth experience and education level, while Model 2 was established to investigate menstrual experience. Childbirth experience was negatively associated with cycle management level. In women with childbirth experience, the odds of having a certain cycle management level decreased by approximately 9.8%. Different education levels were compared with the baseline data (high school level). College-level and University-level education were associated with increased odds of having a certain cycle management level, with odds ratios of 1.119 and 1.122, respectively. However, graduate school level did not show a significant effect on cycle management level.

**Table 5:** Results of statistical analyses using logistic regression to determine likelihood of period tracking app (PTA) usage.

Variables				Crude OR^a^		95% CI^a^		*P* value^a^		Adjusted OR^b^		95% CI^b^		*P* value^b^	
**Model 1 social factor**															
	Childbirth Experience			0.942		0.882-1.002		.06		0.902		0.809-0.995		.04	
	**Education level**														
		Baseline (High school graduate)	—^c^		—		—		—		—		—		
		College level	1.122		1.020-1.225		.02		1.119		1.015-1.224		.03		
		University level	1.129		1.038-1.221		.006		1.122		1.028-1.216		.01		
		Graduate school level	1.094		0.967-1.221		.15		1.071		0.940-1.201		.29		
**Model 2 menstrual experience**															
	Period irregularity			0.928		0.905-0.951		<.001		0.929		0.903-0.954		<.001	
	Number of PMS^d^ symptoms			1.007		0.989-1.025		.45		1.006		0.987-1.024		.54	
	Number of Dysmenorrhea symptoms			1.024		1.010-1.038		.001		1.024		1.010-1.039		.001	

^a^Crude odds ratio value from univariate model.

^b^Multivariate model. Adjusted odds ratio using the following predictors: age, marriage, menarche age, area group, visit obstetrics and gynecology in 3 years and occupation.

^c^Not applicable.

^d^PMS: premenstrual syndrome.

Among the menstrual experience variables, period irregularity and number of dysmenorrhea symptoms had statistically significant effects on the cycle management level (with 95% confidence). When period irregularity increased by one level, the odds of reaching a certain cycle management level decreased by approximately 0.929. In contrast, the number of dysmenorrhea symptoms and cycle management levels were positively associated with an odds ratio of 1.024. (Ordinal logistic regression results are shown in Table S5 in Multimedia Appendix 5).

### Results of the FGIs

FGIs were conducted among eight participants and their responses were recorded and coded. The profiles of interview participants can be found in Table S6 in Multimedia Appendix 6. The participants’ comments and opinions revealed four major themes and fourteen subthemes as follows: (I) menstruation management: (1) self-calendar, (2) pain management, and (3) app use; (II) barriers to following the cycle management: (4) period irregularity, and (5) childbirth and care; (III) motivators to use apps: (6) dysmenorrhea, (7) pregnancy plan, (8) reminders, (9) reference to doctor meeting, (10) set/adjust schedule (11) self-health check; (IV) barriers to continued use of PTAs: (12) lack of app literacy, (13) lack of app accuracy, and (14) time and update issue (Table 6).

**Table 6:** Results of focus group interviews in thematic analysis.

Theme		Quote
**Menstruation management**		
	Self-calendar	*“I don't record it separately since it's a normal cycle, but sometimes it's difficult when I go out without sanitary pads. I sometimes note it in my Naver calendar.”* [NU2, Unmarried] *“I used to record the first date of period in my diary.”* [NU1, 34, Married] *“Ever since I was in high school, my mother has told me to check my calendar myself. So, I’ve been checking it since then."* [NU4, 39, Married] *“I know when I need to take medicine so I can prepare in advance, so I guess I type it into my diary.”* [NU3, 33, Unmarried]
	Pain management	*“I carry two hot packs and put one on my stomach and one on my back…and of course I also taking pain killers.”* [NU3, 33, Unmarried] *“I heard that Omega 3 is good, so I am taking it.”* [NU3, 33, Unmarried] *“I usually take about three 400mg pills of Naproxen-type menstrual pain reliever.”* [NU3, 33, Unmarried] *“I took painkillers for migraine and also get a shot for relieve migraine.”* [NU4, 39, Married] *“I usually take Supplement known for women health such as Vitamin B, evening primrose oil and take pills only when menstruation cramp is severe.”* [AU3, 26, Unmarried] *“When I have menstrual cramps, I just take one or two of ibuprofen pill and it gets better.”* [AU1, 25, Married]
	App use	*“I’m using a Bom calendar to manage my cycle.”* [AU1, 25, Married] *“I am using My calendar (app name).”* [AU2, 34, Married] *“I am using Samsung Health.”* [AU3, 26, Unmarried] *“I use two applications, one called p-tracker, an app provided by iOS, and one that checks cycles on the Apple Health app.”* [AU4, 36, Unmarried]
**Barriers to follow the cycle management**		
	Irregular menstrual cycle (Period irregularity)	*“I also have severe menstrual irregularity, and my periods may be delayed by two or three months. Therefore, I was unable to follow up my cycle management.”* [NU1, 34, Married] *“My friend has an irregular menstrual cycle, and she always carries sanitary pads and medicines with her 365 days a year. That is why she doesn't bother with cycle management.”* [NU3, 33, Unmarried] *“The reason for recording the cycle in this way is that the purpose is to know the due date, but in the case of people who has irregular menstrual cycle, even if they know the due date, there may be cases where they do not menstruate at that time, so the need for cycle management is probably less than that of people with regular period.”* [NU2, 26, Unmarried]
	Childbirth and Care	*“After giving birth, my interest in cycle management is a bit loose. Since I was focused on raising a child, I became less concerned about menstruation than before marriage.”* [NU4, 39, Married] *“My menstrual regularities were so bad that I thought about whether I could get pregnant, so I paid a lot of attention on cycle management before giving birth. Now that I have one child, I really don't manage my menstrual cycle.”* [NU1, 34, Married] *”I spend most of my days caring for my children. Thus, I don't have sufficient time to spend taking care of my cycle management and general health."* [NU4, 39, Married]
**Motivators to adopt period track apps**		
	Dysmenorrhea	*“I visited a gynecologist because my menstrual pain was quite severe. The doctor just informed me that, if my cycle is normal, I should start taking painkillers the day before my period. So, I started using an app to estimate my period and prepare for drugs in advance.”* [AU2, 34, Married] *“I used to have terrible menstrual cramps. Therefore, I was unable to fulfill my planned activities or attend class. So, I looked at the menstrual cycle app to predict my next cycle as well as make a proper schedule.”* [ AU2, 34, Married]
	Pregnancy plan	*“I mentioned it earlier, it will be useful to consult it now in order to confirm the fertility window by ovulation period for all pregnancy planning.”* [AU2, 34, Married] *“I used to use a menstrual cycle app rarely. But now that I'm married and preparing for pregnancy, So, I use it more often.”* [AU2, 34, Married]
	Reminders	*“The app is very useful because it reminds me at the time of period”* [AU4, 36, Unmarried]
	Reference to doctor meeting	*“I set my annual health checkup schedule with gynecologist based on expected cycle in the app”* [AU4, 36, Unmarried] *“It is helpful when I talk with gynecologist”* [AU1, 25, Married]
	Set/adjust schedule	*“As for the advantage of using the app…I think it may be that it is easier to adjust the schedule. I can avoid Important schedules during the period.”* [AU4, 36, Unmarried]
	Self-health check	*“In that app, I can now record details such as my weight and height, as well as my health status. So, I can see how my weight has changed over time, and I think it's great to be able to see in depth of the physical changes too.”* [AU1, 25, Married] *“I can notice my body condition is not good when the cycle became irregular.”* [AU3, 26, Unmarried] *“I could notice my emotion is unstable because of hormone in premenstrual period”* [AU4, 36, Unmarried]
**Barriers to continued use of period track apps**		
	Lack of app literacy	*“Some app features are complex. And there is an app need to touch every single day menstruation is in progress. That is inconvenient.”* [AU4, 36, Unmarried]
	Lack of app accuracy	*“I tried to use the app but quit to use because of app’s inaccuracy to predict next cycle”* [NU3, 33, Unmarried]
	Time and update issue	*“I often forget to record my cycle. So, I delete the app”* [NU2, 26, Unmarried] *“I could not follow up the app use. Because I don’t have much time and concentration”* [NU1, 34, Married] *“I have no complaints about how to use it or the UI, but sometimes, when updates are made or something like that, the menstruation category disappears, which happens once or twice a year. I keep inquiring about things like that and I'm trying to resolve them somehow by updating.”* [AU3, 26, Unmarried]

### PTA User Experience

In total, 431 participants used PTAs as a tool to record their menstrual cycle. More than two-thirds (n=290, 67.3%) of the PTA users have been app users for more than 3 years, and 116 (26.9%) participants have been app users for more than 7 years. The most frequently used application was the Pink diary, which is an official PTA accredited by the Korean Association of Obstetricians and Gynecologists, and 103 (23.9%) respondents were using this app [28]. In addition, a few respondents (n=65, 15%) reported using a period tracker function embedded in their mobile phone health applications, such as Apple Health and Samsung Health (Table S7 in Multimedia Appendix 7).

**Figure 2:**
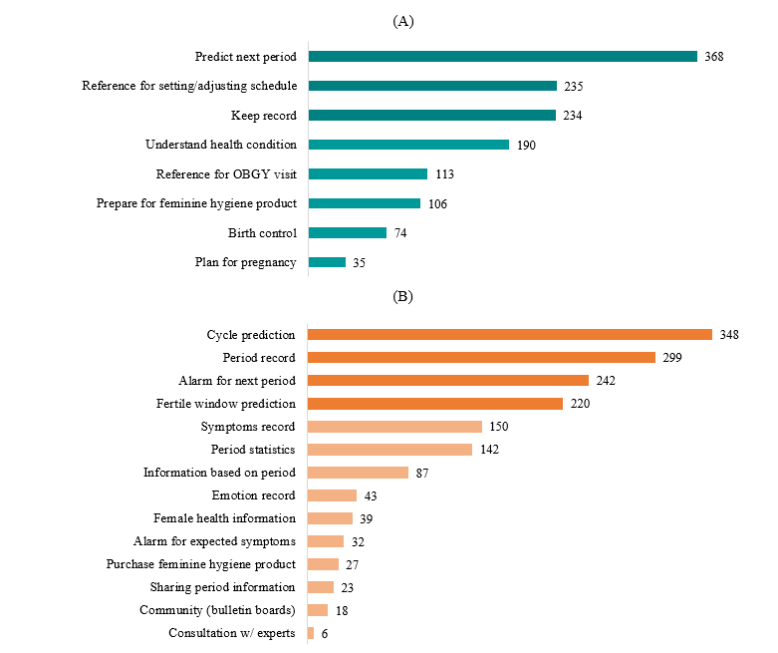
Reasons for using period tracker apps (A) and frequently used features in period tracker apps (B). OBGY: obstetrics and gynecology.

Figure 2 shows the reasons for using PTAs. Most of the app users (n=368, 85%) used the app to predict their next cycle. More than half of the app users used a PTA to keep record (n=235, 54.5%) and to set or adjust their schedule (n=234, 54.3%). The most frequently used feature of PTAs was cycle prediction; 348 (80%) app users used cycle prediction features in their app and period records, while more than half of the respondents used the next period alarm and fertile window prediction features.

**Figure 3:**
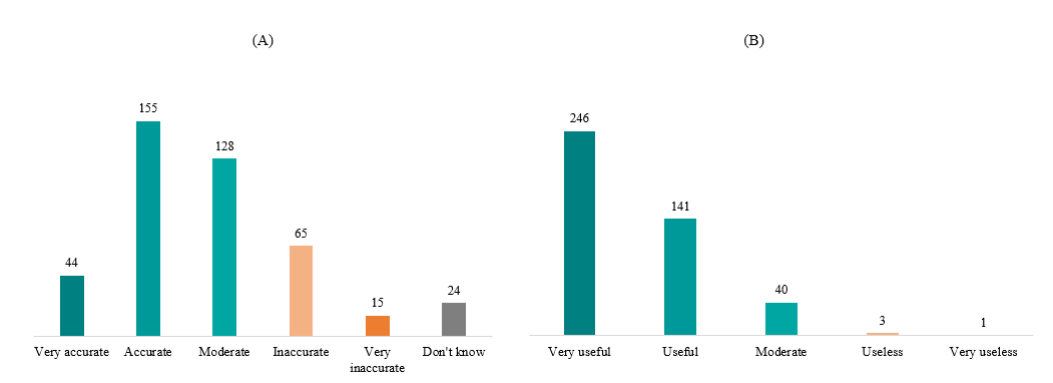
Users' response about predictive accuracy (A) and usability (B) of the period tracker app.

We asked about the accuracy of the cycle prediction and the utility level of the app; 199 (46.2%) app users replied that their app predicted the next cycle with a 2-day error range more than 10 times a year. Some of the respondents who answered that their app was inaccurate explained that this is because of their irregular menstrual cycle. Regarding PTA utility, 387 app users (90%) considered PTAs useful. PTAs played an important role in the healthy menstrual management of the app users in this study (Figure 3).

Using apps is always basic thing when doing something. Basically, I think I should be aware of my health. Women should keep records.

AU2

PTA account for 80point out of 100(in my menstrual management). Menstrual information is very critical and only information to determine my body condition and uterine health so that period tracking is very important.

AU3

In total, 91 (28%) app users complained about seeing too many advertisements on the app. Advertisement is related to the price policy of the apps. Most PTAs provide their apps free of charge to use the main feature and adopt an in-app charge policy to use additional features or eliminate advertisements (Figure S1 in Multimedia Appendix 8). Similarly, most health applications often display pertinent information and commercial ads that enable users to monitor and manage their own health [15,19].

In this study, 414 app users (96.1%) use their apps free of charge. Under these circumstances, it is difficult for app companies to invest in and improve their apps. However, app users are willing to pay when the app has improved accuracy in cycle prediction and features.

If it's a subscription concept, I'm willing to pay 1,000 won per month. so, if it's between 10,000 and 12,000 won per year, I'm willing to pay if I get satisfactory usability from the app.

AU4

If I'm really satisfied with the app, I think I'll pay about 20,000-30,000 won a year to use it.

AU3

It costs less than 10,000 won to download, and if it is really accurate and helpful, I think I will get it.

AU2

If it's a good app, I’d like to spend around 10,000 won per year, and if it's a one-time cost, I'll probably spend up to around 16,000 won.

AU1

## Discussion

### Principal Findings

This study aimed to investigate (1) menstrual cycle management and app usage status, (2) factors affecting PTA usage, (3) factors affecting period tracker cycle management, and (4) motivators and barriers to using PTAs, in millennial and gen Z women.

We found that PTAs are widely used among young women—millennials and gen Z—in South Korea and play an important role in menstrual cycle management. Approximately 90% of the women recorded their menstrual cycles regularly, and over 60% of the respondents used PTA.

The social factors influencing PTA usage included childbirth experience, while menstrual experience factors included the number of PMS symptoms, the number of dysmenorrhea symptoms, and the cycle management level. After childbirth, women were less likely to use PTAs to manage their cycles because of childcare and no concerns about infertility. In addition, women who have given birth tend to neglect their own health management due to childcare responsibilities, so if an app is released that allows them to record postpartum health management and health management according to their child's growth, and receive information, it is expected to further enhance user convenience. To better serve postpartum users, the app should include features tailored to their needs, such as tracking postpartum symptoms, fertility awareness, and contraceptive management. By addressing the specific needs of this demographic, the app can enhance its relevance and usefulness.

Regarding education level, college- and university-level education were positively associated with cycle management level, consistent with the finding that women with higher education levels are likely to manage their cycle more thoroughly compared with those with high school level education. Previous studies have reported that PMS and dysmenorrhea has a negative impact on young women’s education and quality of life [29-38]. It was also consistent with previous research showing that people with dysmenorrhea have higher rates of mental health issues, including anxiety and depression [39], a lower quality of life [4,40-42], and a higher lifetime risk of developing comorbid pain conditions [43]. Another study indicated that adolescent girls' education is affected by dysmenorrhea because of the lack of proper hygiene during menstruation, leading to a higher proportion of dropouts [39]. Therefore, cycle management is an important aspect of women’s education. The more menstrual symptoms a woman experiences, the more diligent she tends to be at managing her cycle. For student users, PTA should include educational tips to help them understand the implications of their symptoms and manage them effectively. This could cover topics such as PMS, dysmenorrhea, and effective cycle management strategies, tailored to accommodate academic schedules.

Our study findings reported that the period irregularity was negatively associated with cycle management level. Women with irregular cycle were less likely to manage their menstrual cycle because of their unpredictable cycle, causing them to prepare for their period every day and to give up on predicting their period. Consistent with previous research on PTA, women with irregular menstrual cycles often find the app to be unhelpful due to its inaccuracies [9]. The existing research offers minimal information on which monitoring capabilities of cycle management that are most crucial for menstruating app users, even though tracking cycle and regularity differed throughout apps [19,21,22]. Thus, it is important to include features that allow users to customize and adapt the app's predictions based on their unique cycle patterns. This could include flexible tracking options for users with irregular cycles, predictive adjustments based on past patterns, and alerts for irregularities. Enhanced algorithms that accommodate a range of cycle irregularities could also improve user experience.

The PTA is used by most app users to predict the next cycle, keep cycle records, and set or change schedules. Regarding the accuracy of PTAs in predicting the next cycle, less than half of app users reported that it was highly accurate. Regardless of the level of accuracy, nearly 90% of app users said that PTAs were useful for menstrual cycle management.

### Strength of the Study

This study has several strengths. First, it is the first study to investigate PTA usage in South Korea. In this study, we elucidated current menstrual management behaviors among millennials and gen Z women. Second, this is the first study to identify social and experiential factors that influence PTA usage and menstrual cycle management. Third, a mixed methods approach was employed, combining quantitative data analysis with qualitative insights gathered from FGIs with the survey participants. This approach allowed for a deeper understanding of the results and provided participants with the opportunity to discuss the data analysis outcomes and share their experiences in detail.

### Limitations

This study has certain limitations. First, there are no universally agreed-upon criteria for evaluating the accuracy of PTAs in predicting the next menstrual cycle. We used arbitrary criteria in the survey to simplify participants' choices. Future studies should focus on developing standardized evaluation criteria for PTAs. Second, we conducted FGIs with only 2 groups (app user and nonuser) covering eight women which may not capture entire gamut of issues related to cycle management and PTAs. Third, owing to privacy concerns, we were unable to contact interviewees for follow-up questions after the FGIs. In future research, providing an opportunity for follow-up questions is essential to validate findings and gather additional insights. Fourth, there are potential biases introduced by recruiting participants from a panel, noting that they may not be representative of the general population and may have a particular interest in menstrual health.

### Conclusions

In conclusion, PTAs are becoming the new normal among millennials and gen Z for managing their menstrual cycles. The use of a PTA empowers women by helping them gain a better understanding of their bodies, ultimately enhancing their social, academic, and health-related lives. Improving the accuracy and literacy of the app is an ongoing task for period-tracking apps, and it is important to provide added value tailored to users' needs. Therefore, the findings of this study should be considered when designing or upgrading PTAs to facilitate the adoption of digital technology among young women, thereby promoting their overall well-being and reproductive health.

## Appendix

Multimedia Appendix 1 Survey information sheet.

Multimedia Appendix 2 Checklist for Reporting Results of Internet E-Surveys (CHERRIES).

Multimedia Appendix 3 Focus group interview guide.

Multimedia Appendix 4 Consolidated criteria for reporting qualitative research (COREQ): a 32-item checklist for interviews and focus groups.

Multimedia Appendix 5 Results of statistical analyses using ordinal logistic regression to determine factors to affect cycle management.

Multimedia Appendix 6 Focus group interview participants profile.

Multimedia Appendix 7 Information of period tracker application users.

Multimedia Appendix 8 App users' complaints when using the period tracker app, non–app user’s reasons not to use the period tracker app, and improvements needed to make nonusers willing to use the period tracker app.

## Abbreviations

CHERRIES: Checklist for Reporting Results of Internet E-Surveys
COREQ: consolidated criteria for reporting qualitative research
FGI: focus group interview
Gen Z: generation Z
PMS: premenstrual syndrome
PTA: Period Tracker App

## Data Availability

The datasets generated and analyzed in the current study are available from the corresponding author upon reasonable request.
