# Supplementary material for: Menstrual Cycle Management and Period Tracker App Use in Millennial and Generation Z Individuals: Mixed Methods Study
Source: J Med Internet Res. 2024 Oct 10;26:e53146. doi: 10.2196/53146 (PMC11502972; doi:10.2196/53146)
Supplement: Multimedia Appendix 1 [file jmir_v26i1e53146_app1.docx]

**Supplementary Table 1. Survey information sheet**

Hello, my name is Minji Hong, and I am currently a Ph.D. candidate in the Department of Medical Device Industry at Yonsei University College of Medicine.

I am conducting a study on the status of women's health management through menstrual cycle management and tracking applications among women of the MZ generation. The survey targets 700 women in their 20s and 30s across the nation.

This survey consists of a maximum of 23 questions, including basic respondent information, questions related to menstrual cycles, and the usage status of cycle tracking apps. It will take approximately 5 minutes to complete.

Through the results of this survey, we aim to understand the menstrual experiences, cycle management methods, and application usage of MZ generation women to deepen our understanding of women's health management.

Participants will be compensated with a noticed amount of Obey money. Participation can be discontinued at any time without any disadvantage. No personal information will be collected in this survey.

For any inquiries related to this research, please contact:

**Researcher Contact Information**: Minji Hong, Ph.D. Candidate, Department of Medical Device Industry, Yonsei University (mj.hong@yonsei.ac.kr)

**For Information on Research Subject Rights**: Yonsei University Institutional Review Board (02-2123-5143)

If you have understood the explanation above and agree to participate in this study, please click "Start Survey" to begin.
