# Supplementary material for: Menstrual Cycle Management and Period Tracker App Use in Millennial and Generation Z Individuals: Mixed Methods Study
Source: J Med Internet Res. 2024 Oct 10;26:e53146. doi: 10.2196/53146 (PMC11502972; doi:10.2196/53146)
Supplement: Multimedia Appendix 2 [file jmir_v26i1e53146_app2.docx]

# **Supplementary Table 2. Checklist for Reporting Results of Internet E-Surveys (CHERRIES)**

| Checklist Item | | Answer |
| --- | --- | --- |
| **Design** | | |
|  | Describe survey design | This study uses a purposive sample. We sent survey invitations to females aged 20-30 |
| **IRB (Institutional Review Board) approval and informed consent process** | | |
|  | IRB approval | The study protocol was reviewed and approved by the Institutional Review Board of Severance Hospital (No. 4-2023-0097). |
|  | Informed consent | The subjects were informed of the purpose of the survey, confidentiality, and voluntary participation principles before responding. |
|  | Data protection | Personal information was only known to the person collecting the data. We provided an anonymous database for analysis by researchers. The collected data was stored on a specific computer server in the healthcare management lab at Yonsei University. |
| **Development and pre-testing** | | |
|  | Development and testing | Respondents’ data were collected via the Ovey application. Before formal data collection, a preliminary sample tested the survey procedure, and the questionnaire was revised based on their feedback. |
| **Recruitment process and description of the sample having access to the questionnaire** | | |
|  | Open survey versus closed survey | Closed survey. Only a registered panel of females aged 20-30 was invited to the survey. |
|  | Contact mode | Initial contact was made via application push message. |
|  | Advertising the survey | \| No advertisement was used. \| \| --- \| |
| **Survey administration** | | |
|  | Web/E-mail | The survey was published on a mobile survey application (Ovey). |
|  | Context | Ovey(Open Survey) is a platform that can provide surveys and pay compensation and has 830,000 voluntarily registered panels. |
|  | Mandatory/voluntary | The survey was voluntary. |
|  | Incentives | App points (worth about 500 Korean won) were provided for completed participants. |
|  | Time/Date | Data was collected on March 27, 2023. |
|  | Randomization of items or questionnaires | To prevent selection biases, some questionnaire items were randomized. |
|  | Adaptive questioning | There were adaptive questions in the questionnaire, allowing respondents to skip unnecessary questions based on their answers. For example, if the subjects answered that they had not used a period tracker app, then period tracker app user experience questions were skipped. |
|  | Number of Items | The survey had between 15 and 26 questions. |
|  | Number of screens (pages) | 15-26 screens (mobile pages). Each question was shown on one page. |
|  | Completeness check | Technically, only completed surveys could be submitted. |
|  | Review step | Respondents were not allowed to go 'back' in the survey. |
| **Response rates** | | |
|  | Unique site visitor | N/A. |
|  | View rate (Ratio of unique survey visitors/unique site visitors) | The survey is voluntary. The system cannot record the number of visitors, so the view rate cannot be calculated. |
|  | Participation rate (Ratio of unique visitors who agreed to participate/unique first survey page visitors) | The system cannot record the number of people who filled in the survey and the number of visitors, so the participation rate cannot be calculated. |
|  | Completion rate (Ratio of users who finished the survey/users who agreed to participate) | This was a voluntary questionnaire. By default, participants submit questionnaires on behalf of their consent to participate, so the completion rate cannot be calculated. |
| **Preventing multiple entries from the same individual** | | |
|  | Cookies used | N/A |
|  | IP check | N/A |
|  | Log file analysis | N/A |
|  | Registration | One Panel ID could participate in the survey only once. |
| **Analysis** | | |
|  | Handling of incomplete questionnaires | Only completed questionnaires were analyzed. |
|  | Questionnaires submitted with an atypical timestamp | We did not exclude subjects based on the time taken to complete the survey. |
|  | Statistical correction | Logistic regression and a generalized linear model (with log link and gamma distribution) were used. |
