# Supplementary material for: Menstrual Cycle Management and Period Tracker App Use in Millennial and Generation Z Individuals: Mixed Methods Study
Source: J Med Internet Res. 2024 Oct 10;26:e53146. doi: 10.2196/53146 (PMC11502972; doi:10.2196/53146)
Supplement: Multimedia Appendix 3 [file jmir_v26i1e53146_app3.docx]

**Supplementary Table 3. Focus Group Interview Guide**

**PTA User Group**

| **Introductory Questions** |
| --- |
| What is the most inconvenient aspect of menstruation? |
| How do menstrual pain or premenstrual syndrome (PMS) affect your daily life? |
| **Core Questions** |
| 1. What motivated you to use the period tracking app? |
| 2. How important do you think the period tracking app is for women’s health management? |
| 3. What are the advantages of menstrual management through the period tracking app? |
| 4. Are you satisfied with the accuracy of the app's cycle predictions? |
| 5. What features of the period tracking app do you find useful? |
| 6. Why do those who have experienced childbirth tend to manage their cycles less effectively? (Did the method or degree of cycle management change before and after childbirth?) |
| 7. The survey indicates that the more irregular the cycle, the less it is managed. Why do you think this is the case? |
| 8. The more severe the symptoms of menstrual pain or PMS, and the greater the impact on daily life, the more diligently people tend to manage their cycles. What is your experience in this regard? |
| 9. If you have experienced impacts on your studies due to menstrual pain or PMS, please share. |
| **Concluding Questions** |
| If the accuracy or functionality of the period tracking app improved, how much would you be willing to pay annually? |
| What improvements or additional features would you like to see in the period tracking app? |
| What do you think is most necessary to improve the menstrual experience? |

*Questions can be added or modified based on participants' responses.

**Non-User Group**

| **Introductory Questions** |
| --- |
| What is the most inconvenient aspect of menstruation? (Discuss your experiences with menstrual pain and PMS.) |
| How do menstrual pain or premenstrual syndrome (PMS) affect your daily life? |
| How do you usually manage menstrual pain or PMS? |
| **Core Questions** |
| 1. How do you manage your menstrual cycle? |
| 2. Have you been treated for menstrual irregularities or other female disorders? (If yes, has your management method changed before and after treatment?) |
| 3. What do you know about period tracking apps?  (Provide a brief explanation of the app if necessary.) |
| 4. If you have used any apps, please mention their names. |
| 5. What are the reasons for not using or discontinuing the period tracking app? |
| 6. Would you consider using a period tracking app if certain features were improved or added? |
| 7. What are your concerns about privacy breaches in period tracking apps? |
| 8. If period tracking apps were linked to medical consultations or offered telemedicine services, would you be willing to use them? |
| 9. Why do those who have experienced childbirth tend to manage their cycles less effectively? (Did the method or degree of cycle management change before and after childbirth?) |
| 10. The survey indicates that the more irregular the cycle, the less it is managed. Why do you think this is the case? |
| 11. The more severe the symptoms of menstrual pain or PMS, and the greater the impact on daily life, the more diligently people tend to manage their cycles. What is your experience in this regard? |
| 12. If you have experienced impacts on your studies due to menstrual pain or PMS, please share. |
| **Concluding Questions** |
| If the accuracy or functionality of the period tracking app improved, how much would you be willing to pay annually? |
| What do you think is most necessary to improve the menstrual experience? |

*Questions can be added or modified based on participants' responses.
