# Supplementary material for: Menstrual Cycle Management and Period Tracker App Use in Millennial and Generation Z Individuals: Mixed Methods Study
Source: J Med Internet Res. 2024 Oct 10;26:e53146. doi: 10.2196/53146 (PMC11502972; doi:10.2196/53146)
Supplement: Multimedia Appendix 4 [file jmir_v26i1e53146_app4.docx]

# **Supplementary table 4. Consolidated criteria for reporting qualitative research (COREQ): a 32-item checklist for interviews and focus groups**

| Item | | Answer |
| --- | --- | --- |
| **Personal Characteristics** | |  |
|  | 1.Interviewer/facilitator | First author Minji Hong has conducted the interview |
|  | 2.Credentials | MA in Economics, Market researcher |
|  | 3.Occupation | Researcher & PhD student in Yonsei University |
|  | 4.Gender | Female |
|  | 5.Experience and training | She has worked as a market researcher in public and private research institutes for 5 years and has extensive experience in conducting interviews, particularly group interviews. |
| **Relationship with participants** | |  |
|  | 6.Relationship established | No prior relationship with the participants. |
|  | 7.Participant knowledge of the interviewer | Participants were provided with personal profiles, reasons for conducting the research, and a research brief. |
|  | 8.Interviewer characteristics | The interviewer’s reasons and interests in the research topic were shared with the participants. |
| **Theoretical framework** | |  |
|  | 9.Methodological orientation and Theory | Mixed method-explanatory sequential design |
| **Participant selection** | |  |
|  | 10.Sampling | Purposive sampling. |
|  | 11.Method of approach | Online meetings (face-to-face). |
|  | 12.Sample size | 8 participants. |
|  | 13.Non-participation | One person refused to participate due to a personal matter before the interview and was replaced by another candidate. |
| **Setting** | |  |
|  | 14.Setting of data collection | Conducted in a dry lab. |
|  | 15.Presence of non-participants | Yes, an employee of the survey company was present to support technical issues. |
|  | 16.Description of sample | Participants were divided into two groups—period tracker users and non-users. Key selection factors included age and marital status. |
| **Data collection** | |  |
|  | 17.Interview guide | Questions were provided by the author, with additional questions asked based on participants’ responses. |
|  | 18.Repeat interviews | Each group was interviewed once. |
|  | 19.Audio/visual recording | Visual recording was conducted with participants' prior consent. |
|  | 20.Field notes | Field notes were made during the interviews. |
|  | 21.Duration | Each interview lasted about 60 minutes. |
|  | 22.Data saturation | Data saturation was discussed among the researchers. |
|  | 23.Transcripts returned | Transcripts were not returned to participants for verification. |
| **Data analysis** | |  |
|  | 24.Number of data coders | 2 coders. |
|  | 25.Description of the coding tree | Yes, a coding tree was developed. |
|  | 26.Derivation of themes | Some themes were identified in advance, while others were derived from the data after the interviews. |
|  | 27.Software | NVivo software 14 was used. |
|  | 28.Participant checking | No participant checking was conducted. |
| **Reporting** | |  |
|  | 29.Quotations presented | Participant quotations were presented to illustrate the findings. |
|  | 30.Data and findings consistent | There was consistency between the data presented and the findings. |
|  | 31.Clarity of major themes | Major themes were clearly presented in the findings. |
|  | 32.Clarity of minor themes | Minor themes were discussed in the findings. |
