# Supplementary material for: Menstrual Cycle Management and Period Tracker App Use in Millennial and Generation Z Individuals: Mixed Methods Study
Source: J Med Internet Res. 2024 Oct 10;26:e53146. doi: 10.2196/53146 (PMC11502972; doi:10.2196/53146)
Supplement: Multimedia Appendix 5 [file jmir_v26i1e53146_app5.docx]

# **Supplementary Table 5. Results of statistical analyses using ordinal logistic regression to determine factors to affect Cycle management.**

|  |  | **Variables** | **Adjusted**  **OR^a^** | **Estimate** | ***p-value*** | **95% CI** |
| --- | --- | --- | --- | --- | --- | --- |
| **Model1 Social Factor** | | | | | | |
|  | Childbirth Experience | | 0.592 | -2.00 | .046 | 0.354 – 0.991 |
|  | **Education level** | |  |  |  |  |
|  |  | Baseline (High school graduate) | |  |  |  |
|  |  | College level | 1.941 | 2.28 | .02 | 1.097 – 3.433 |
|  |  | University level | 1.933 | 2.54 | .01 | 1.163 – 3.210 |
|  |  | Graduate school level | 1.451 | 1.04 | .30 | 0.720 – 2.923 |
| **Model2 Menstrual experience** | | | | | | |
|  | Period irregularity | | 0.664 | -5.55 | <.001 | 0.574 – 0.767 |
|  | Number of PMS symptoms | | 1.038 | 0.70 | .49 | 0.934 – 1.153 |
|  | Number of Dysmenorrhea symptoms | | 1.147 | 3.37 | .001 | 1.060 – 1.249 |

a. Result was adjusted by following predictor: age, marriage, menarche age, area group, visit OBGY in 3years and occupation.
