# Supplementary material for: Menstrual Cycle Management and Period Tracker App Use in Millennial and Generation Z Individuals: Mixed Methods Study
Source: J Med Internet Res. 2024 Oct 10;26:e53146. doi: 10.2196/53146 (PMC11502972; doi:10.2196/53146)
Supplement: Multimedia Appendix 6 [file jmir_v26i1e53146_app6.docx]

# **Supplementary Table 6. Focus Group Interview Participants Profile**

| **Group** | **ID** | **Panel Code** | **Age** | **Generation** | **Marriage**  **Status** | **No. of Child** | **Cycle**  **Management** |
| --- | --- | --- | --- | --- | --- | --- | --- |
| App User | AU1 | fa30404 | 25 | Z | Married | None | PTA |
| App User | AU2 | d9cdbd0 | 34 | M | Married | 2 | PTA |
| App User | AU3 | abbd542 | 26 | Z | Unmarried | None | PTA |
| App User | AU4 | 18c0dc6 | 36 | M | Unmarried | None | PTA |
| Non-User | NU1 | 23cc81c | 34 | M | Married | 1 | Diary |
| Non-User | NU2 | 26df49d | 26 | Z | Unmarried | None | Calendar |
| Non-User | NU3 | 8125bc9 | 33 | M | Unmarried | None | Calendar |
| Non-User | NU4 | c2ebe48 | 39 | M | Married | 1 | Diary, Calendar |

Abbreviation: PTA-Period Track Application, AU:App user, NU:Non-user, M:Millenials, Z:GenZ
