# Supplementary material for: Menstrual Cycle Management and Period Tracker App Use in Millennial and Generation Z Individuals: Mixed Methods Study
Source: J Med Internet Res. 2024 Oct 10;26:e53146. doi: 10.2196/53146 (PMC11502972; doi:10.2196/53146)
Supplement: Multimedia Appendix 7 [file jmir_v26i1e53146_app7.docx]

# **Supplementary Table 7. Information of period tracker application users**

| Category | | n (%) |
| --- | --- | --- |
| **App usage period** | |  |
|  | Less than 1year | 31(7.2) |
|  | 1 year - <2years | 41(9.5) |
|  | 2 year - <3years | 69(16.0) |
|  | 3 year - <5years | 94(21.8) |
|  | 5 year - <7years | 80(18.6) |
|  | More than 7 years | 116(26.9) |
| **Period tracker app name** | |  |
|  | Pink Diary | 103(23.9) |
|  | Heymoon | 52(12.1) |
|  | Apple Health | 50(11.6) |
|  | Bom calendar | 36(8.4) |
|  | Flo | 29(6.7) |
|  | Women Period Calendar | 26(6.0) |
|  | The day | 18(4.2) |
|  | My calendar | 18(4.2) |
|  | Samsung Health | 15(3.5) |
|  | P Tracker | 12(2.8) |
|  | Etc. | 49(11.4) |
|  | Don’t know | 23(5.3) |
| **Total** | | 431(100.0) |
