# Supplementary material for: Menstrual Cycle Management and Period Tracker App Use in Millennial and Generation Z Individuals: Mixed Methods Study
Source: J Med Internet Res. 2024 Oct 10;26:e53146. doi: 10.2196/53146 (PMC11502972; doi:10.2196/53146)
Supplement: Multimedia Appendix 8 [file jmir_v26i1e53146_app8.docx]

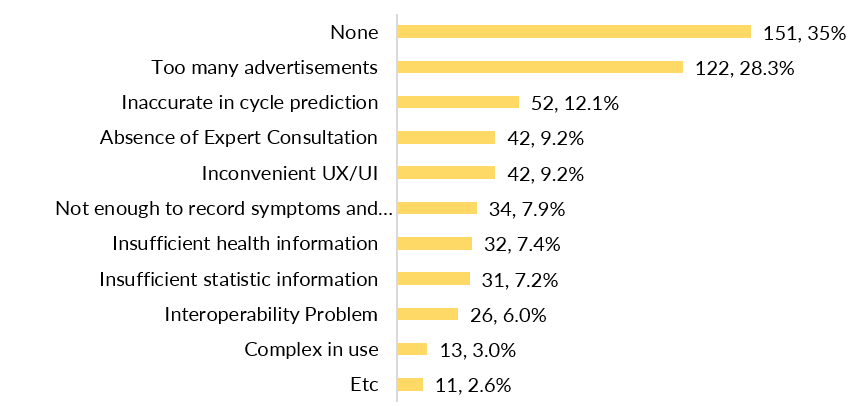
**Supplementary figure 1**

- 1. App users' complaints when they are using the period tracker app.


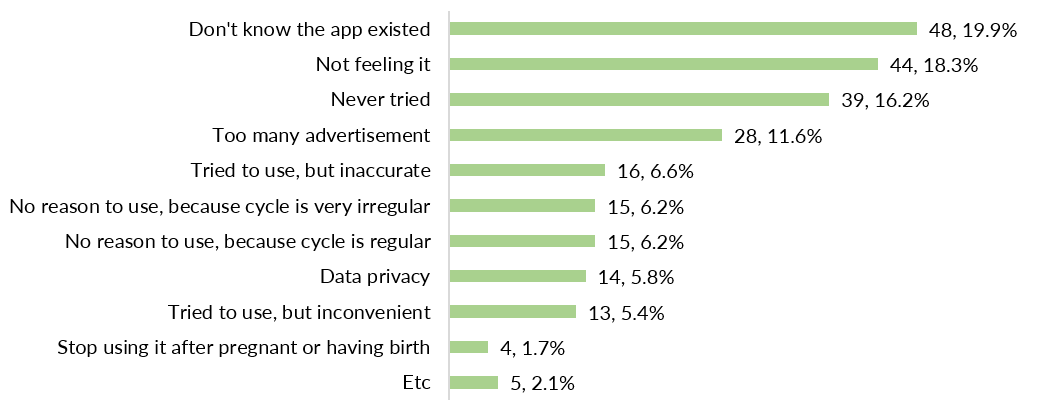
Note: Participants allowed to respond up to 2 answers.

- 1. Non-App user’s reasons not(quit) to use the period tracker app.

Note: Participants allowed to respond only 1 answer.

- 1.
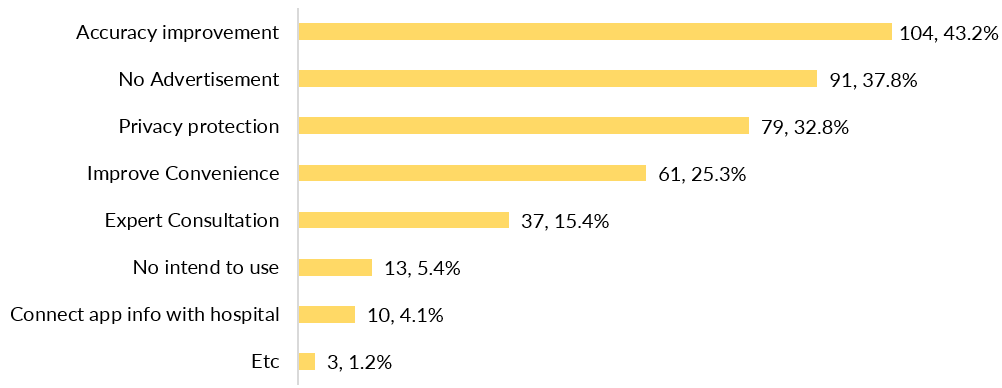
Improvement needs to make non-users willing to use the period tracker app.

Note: Participants allowed to respond up to 2 answers.
